# Supplementary material for: Development of chemokine network inhibitors using combinatorial saturation mutagenesis
Source: Commun Biol. 2025 Apr 3;8:549. doi: 10.1038/s42003-025-07778-6 (PMC11969024; doi:10.1038/s42003-025-07778-6)
Supplement: Supplementary file 4 — Supplementary Data 2 [file 42003_2025_7778_MOESM4_ESM.zip › SupplementaryData3/scripts/xattune_ci.tar!/./script/analysis/JK/JK_SMA_ChemokineSuppliers.docx]

Supplementary Table: Chemokine Suppliers for Cell Migration Experiments

| Chemokine | Supplier | Supplier Code |
| --- | --- | --- |
| CCL1 | Peprotech | 300-37 |
| CCL11 | Peprotech | 300-21 |
| CCL13 | Peprotech | 300-24 |
| CCL14 | Peprotech | 300-38B |
| CCL15 | Novus | NBP2-35043 |
| CCL16 | Peprotech | 300-44 |
| CCL17 | Peprotech | 300-30 |
| CCL18 | Peprotech | 300-34 |
| CCL19 | Peprotech | 300-29B |
| CCL2 | Peprotech | 300-04 |
| CCL20 | Peprotech | 300-29A |
| CCL21 | Peprotech | 300-35A |
| CCL22 | Peprotech | 300-36A |
| CCL23 | Biolegend | 587002 |
| CCL24 | Peprotech | 300-33 |
| CCL25 | Peprotech | 300-45 |
| CCL26 | Peprotech | 300-48 |
| CCL27 | Peprotech | 300-54 |
| CCL28 | Peprotech | 300-57 |
| CCL3 | Peprotech | 300-08 |
| CCL4 | Peprotech | 300-09 |
| CCL4L1 | Peprotech | 300-58 |
| CCL5 | Peprotech | 300-06 |
| CCL7 | Peprotech | 300-17 |
| CCL8 | Peprotech | 300-15 |
| CX3CL1 | Peprotech | 300-31 |
| CXCL1 | Peprotech | 300-11 |
| CXCL10 | Peprotech | 300-12 |
| CXCL11 | Peprotech | 300-46 |
| CXCL12 | Peprotech | 300-28A |
| CXCL13 | Peprotech | 300-47 |
| CXCL14 | Peprotech | 300-50 |
| CXCL16 | Peprotech | 300-55 |
| CXCL2 | Peprotech | 300-39 |
| CXCL3 | Peprotech | 300-40 |
| CXCL4 | Peprotech | 300-16 |
| CXCL5 | Peprotech | 300-22 |
| CXCL6 | Peprotech | 300-41 |
| CXCL7 | Peprotech | 300-14 |
| CXCL8 | Peprotech | 200-08 |
| CXCL9 | Peprotech | 300-26 |
| XCL1 | Peprotech | 300-20 |
